# Biomass of *Spirulina maxima* enriched by biosorption process as a new feed supplement for swine

**DOI:** 10.1007/s10811-012-9901-6

**Published:** 2012-09-25

**Authors:** A. Saeid, K. Chojnacka, M. Korczyński, D. Korniewicz, Z. Dobrzański

**Affiliations:** 1 Institute of Inorganic Technology and Mineral Fertilizers, Wroclaw University of Technology, I-26, ul. Smoluchowskiego 25, 50-372 Wrocław, Poland; 2 Department of Animal Hygiene and Environment, Wroclaw University of Environmental and Life Sciences, ul. Chełmońskiego 38 C, 51-630 Wrocław, Poland; 3 Department of Animal Nutrition and Food Science, Wroclaw University of Environmental and Life Sciences, ul. Chełmońskiego 38 C, 51-630 Wrocław, Poland

**Keywords:** Animal feeding, Swine, Mineral feed additives, *Spirulina maxima*, Copper

## Abstract

This paper deals with the new mineral feed additives with Cu produced in a biosorption process from a semi-technical scale. The natural biomass of edible microalga *Spirulina* sp. was enriched with Cu(II) and then used as a mineral supplement in feeding experiments on swine to assess its nutrition properties. A total of 24 piglets divided into two groups (control and experimental) were used to determine the bioavailability of a new generation of mineral feed additives based on *Spirulina maxima*. The control group was feed using traditional inorganic supplements of microelements, while the experimental group was fed with the feed containing the biomass of *S. maxima* enriched with Cu by biosorption. The apparent absorption was 30 % (*P* < 0.05) higher in the experimental group. No effect on the production results (average daily feed intake, average daily gain, feed conversion ratio) was detected. It was found that copper concentration in feces in the experimental group was 60 % (*P* < 0.05) lower than in the control group. The new preparation—a dietary supplement with microelements produced by biosorption based on biomass of microalgae *S. maxima*—is a promising alternative to currently used inorganic salts as the source of nutritionally important microelements.

## Introduction

In recent years, there has been concern about the accumulation of minerals in the environment, especially copper (Cu) (Dach and Starmans 2005; Zielińska et al. 2009). Pig diets are usually supplemented with Cu in a dose that sometimes largely exceeds the physiological requirements. Most of the dietary supply is excreted. Manure may contain high amounts of Cu (Jondreville et al. 2003) which has the negative environmental impact. An increasing problem associated with intensive swine production is the concentration of swine manure. Despite its fertilizer value, a certain risk of contamination exists. In some areas, swine manure is collected in pits and spread on agriculture land as slurry using tank wagons (Duffera et al. 1999).

Trace minerals have traditionally been supplemented to livestock diets as inorganic salts that have been reported to have low bioavailability (Chowdhury et al. 2004; Miles and Henry 2000). One of the remarkable effects of high level of copper sulfate (CuSO_4_·5H_2_O) in diets for poultry is the gizzard erosion (Chowdhury et al. 2004; Miles and Henry 2000). Improvement of Cu availability may be achieved by providing dietary sources of these microelements in chelated or complexed form, that have shown to be more available form (McDowell 2003). There are various types of organic trace minerals on the market: complexes, amino acid chelates and proteinates (Spears et al. 2004). Amino acid chelates are reported to have significantly higher absorption rates from the intestine as compared to soluble inorganic metal salts (Brady et al. 1978; Ashmead 1985; Ashmead and Zunino 1993; Baker and Ammerman 1995; Bovell-Benjamin et al. 2000). At the same time, it was found that Cu–methionine chelate might carry all the acidic nature of the reactants and therefore cause gizzard erosion, since is manufactured by reacting CuSO_4_·5H_2_O with methionine. Copper ions are known to be a very strong catalyst (Chowdhury et al. 2004; Miles and Henry 2000).

Microalgae have been long valued as food and feed. Algae were also considered as a source of essential bioactive compounds for organisms. They provide nearly all essential vitamins (A, B_1_, B_2_, B_6_, B_12_, C, E, nicotinamide, biotin, folic acid, and panthenoic acid), polyunsaturated fatty acids (GLA, AA, EPA, DHA) and pigments, such as β-carotene or astaxanthin (Spolaore et al. 2006; Dufosse et al. 2005; Marquez-Rocha et al. 1993, 1995). The microalgal industry has gained importance due to its utilization in different field of biotechnological process during the last three decades (Spolaore et al. 2006; Borowitzka 1999; Ohira et al. 2010). Their biosorption abilities were investigated thoroughly (Chojnacka et al. 2004; Abu Al-Rub et al. 2006).

Biosorption is known as selective and effective method of removing pollutants from waste water (Kadukova and Vircikova 2005; Donmez et al. 1999; Aksu 2001). In this study, the mentioned process was applied as technique of binding metal ions to the biomass of microalgae which are nutritionally significant in animals (Zielińska and Chojnacka 2009).

The process of binding microelements to the biomass is based on the ability of biological materials to bind metal ions by either metabolically mediated or purely physico-chemical pathways of uptake (Oporto et al. 2006). Because of negative surface charge and membrane composition, microalgae are natural adsorbents of metal ions (Kargi and Cikla 2006). Cell walls of microalgae, consist mainly of polysaccharides, proteins and lipids and contains many functional groups (such as carboxylate, hydroxyl, thiol, sulfonate, phosphate, amino and imidazole groups) that can form coordination complexes with metal cations (Gong et al. 2005; Yan and Pan 2002) and these functional groups are able to interact with metal ions in an aqueous solution (Saygideger et al. 2005).

The aim of this paper was to evaluate the availability of new generation of mineral feed additives based on microalgae. *Spirulina maxima* biomass that was enriched with Cu(II) by biosorption and then, used as a mineral supplement in feeding experiments on swine to assess its nutritional properties.

## Material and methods

The microalga *Spirulina maxima* was cultivated in a stirred tank reactor (dimensions 1.12 m × 3.6 m) with a capacity 10 m^3^, covered by a glasshouse, equipped with the biomass separation system (six bag filters, average pore size 6 μm, Desjoyaux Co, Ltd.), mixing system (pumps) and six lamps (300 W Astral Pool, Poland). *S. maxima* was obtained from the Culture Collection of Algal Laboratory (CCALA) Institute of Botany, Academy of Sciences of the Czech Republic. Microalga was cultivated in the Schlösser (1982) medium, prepared with technical grade reagents.

### Biosorption experiments

The biomass of *S. maxima* was enriched with copper (II) ions via biosorption. The enrichment processes was performed in containers containing 45 L of metal ions solution at ambient temperature in tap water. The solutions were prepared by dissolving appropriate amounts of inorganic salt CuSO_4_·5H_2_O admitted for using as a source of Cu(II) in animal diets (from POCH, Gliwice, Poland) (Feeding Standards for Poultry and Swine 2005). The contact time was 2 h as determined previously in kinetic experiments (Michalak et al. 2007). After this time, enriched biomass was separated on a filter with 6 μm pore diameter, dried at 50 °C and ground. Initial concentration of metal ions in the solution was *C*_0_ 300 mg L^−1^. pH of the solution was adjusted with NaOH/HCl to pH 5. The biomass concentration was 1 g of dry mass L^−1^.

### Feeding experiments

#### Feed

The standard feed was composed of: wheat, hordeum, canola oil, soybean meal, and specially prepared for each stage of the experiments (starter, grower and finisher) mix diet, similar feed was used elsewhere (Korniewicz et al. 2012) (Tables 1 and 2). The content of nutrients and feed additives is presented in Table 3. The source of vitamins and microelements was a commercially available premix produced by LNB Poland. The experimental group was fed with the same feed but microelements were supplemented by *S*. *maxima* enriched with microelements by biosorption.

**Table 1:** The composition of the diet (mg kg^−1^)

Ingredient	Starter	Grower	Finisher
Metabolic energy, kcal kg^−1^	2,340	2,280	2,280
Total protein	169	156	144
Fiber	330	33.0	34.0
Crude Ash	510	44.0	38.0
Oil and fat	50.0	35.0	32.0
Methionine	3.80	32.0	2.90
Lysine	11.7	94.0	8.10
Tryptophan	2.10	19.0	1.70
Isoleucine	6.60	5.90	5.40
Methionine + cysteine	7.00	6.40	6.00
Threonine	7.30	6.10	5.50
Total calcium	8.10	6.80	5.80
Available phosphorus	3.10	2.50	2.20
Total phosphorus	4.9	4.20	4.00
Total sodium	2.0	1.90	1.60

**Table 2:** Percent composition and feeding value of mixtures for fatteners

Ingredients	Units	Type of mixture		
		Starter	Grower	Finisher
Ground wheat	%	35.0	40.0	40.0
Ground barley	%	41.7	43.4	49.4
Soya bean oilmeal	%	15.5	11.5	6.5
Soya oil	%	3.3	1.8	1.4
Acidifier	%	0.5	0.3	0.2
Supplementary feed Starter	%	4.0	–	–
Supplementary feed Grower	%	–	3.0	–
Supplementary feed Finisher	%	–	–	2.5
Total	%	100	100	100

**Table 3:** The nutrient content of the diets with amino acids of mixtures for fatteners

Ingredients	Units	Type of mixture		
		Starter	Grower	Finisher
Chemical composition, analyzed, per kg of mixture:				
Net energy	kcal	2,340	2,280	2,281
Metabolizable energy	MJ	13.60	13.25	13.25
Dry matter	%	87.3	87.2	87.1
Crude protein	%	17.4	15.7	14.5
Crude fiber	%	3.0	2.8	3.5
Crude fat	%	5.0	3.1	3.2
Crude ash	%	5.1	4.3	3.7
N-free extractives	%	56.8	61.3	62.2
l-Lysine	%	1.17	0.93	0.85
dl-Methionine	%	0.39	0.29	0.26
Methionine + cysteine	%	0.71	0.60	0.55
l-Threonine	%	0.75	0.59	0.54
Tryptophan	%	0.23	0.20	0.16
Isoleucine	%	0.66	0.59	0.51
Ca	%	0.73	0.68	0.60
P total	%	0.55	0.50	0.43
Mineral P	%	0.16	0.15	0.13
Digestible P	%	0.34	0.30	0.25
Phytase	FTU	500	510	425
Na	%	0.20	0.20	0.14
Fe^a^	mg	198	183	172
Mn^a^	mg	91	82	73
Cu^a^	mg	167	25	21.8
Zn^a^	mg	157	148	126
I^a^	mg	1.66	1.49	1.26
Co^a^	mg	0.88	0.81	0.68
Se^a^	mg	0.49	0.48	0.44
Vitamin A^b^	I.U.	16 000	12 000	10 000
Vitamin D3^b^	I.U.	2 000	1 998	1 665
Vitamin E^b^	mg	150.00	124.50	103.75
Vitamin K3^b^	mg	4.00	1.80	1.50
Vitamin B_1_^b^	mg	2.40	1.80	1.50
Vitamin B_2_^b^	mg	6.40	4.80	4.00
Niacin^b^	mg	32.00	24.00	20.00
Pantothenic acid^b^	mg	16.00	12.00	10.00
Vitamin B_6_^b^	mg	4.80	3.60	3.00
Vitamin B_12_^b^	mcg	40.00	30.00	25.00
Biotin^b^	mcg	160.00	120.00	100.00
Vitamin C^b^	mg	100.00	100.00	83.30
Folic acid^b^	mg	3.20	2.40	2.00
Choline^b^	mg	350.00	250.00	208.30

^a^Microelements supplemented: Fe as FeSO_4*_H_2_O 30 %; Mn as MnO_2_ 60 %; Cu as CuSO_4*_5H_2_O 25 %; Zn as ZnSO_4*_H_2_O 35 %; I as Ca(IO_3_)_2*_H_2_O 62 %, Co as CoCO_3_ 21 %; Se as Na_2_SeO_3_ 5 %

^b^Vitamins supplemented: vitamin A (retinyl acetate), vitamin D_3_ (cholecalciferol), vitamin E (dl alpha tocopherol acetate), vitamin K (menadione sodium bisulfite), vitamin B_1_ (thiamine mononitrate), vitamin B_2_ (riboflavin), vitamin B_3_ (nicotinic acid), vitamin B_5_ (d-calcium pantothenate), vitamin B_6_ (pyridoxine hydrochloride), vitamin B_12_ (cyanocobalamin), biotin (d-biotin), vitamin C (ascorbic acid), folic acid (folic acid), choline (choline chloride)

The enriched biomass of *S. maxima* via biosorption process was investigated as the source of microelement—Cu(II), Fe(II), and Zn(II). The content of Cu, Fe, and Zn in the biomass after biosorption process was as follows: 30.6, 46.5, and 31.5 mg g^−1^, respectively. Because biomass enriched with copper also contains also other microelements, Fe and Zn, its content was taken into consideration during planning the experiment. Two experimental groups were distinguished:

Group I The microelements requirement was covered by inorganic salts, Control (C),
Group II The requirement for Cu(II) was covered by *S. maxima* biomass enriched with copper (Sm–Cu) (100 %),
  The requirement for Fe was covered by *S. maxima* biomass enriched with iron (Sm–Fe) (25.5 %) and 74.5 % with inorganic salt.
  The requirement for Zn was covered by *S. maxima* biomass enriched with zinc (Sm–Zn) (17.3 %) and by inorganic salt (82.7 %).

Leeson and Caston (2008) proved that trace minerals are oversupplied in feed formulations. Assuming the enhanced bioavailability of trace minerals in the form of enriched biomass of *S. maxima*, dietary levels of Cu at first stage of experiments (starter—where the level of microelement is the highest) were minimized in experimental group to about 50 % in the comparison to the control group (Table 4).

**Table 4:** The content of control and experimental feed for pigs (mg kg^−1^ ± measurement uncertainty)

		Starter		Grower		Finisher	
		Control	Experimental	Control	Experimental	Control	Experimental
Microelements	Cu	13.3 ± 2.0	6.57 ± 1.64	9.06 ± 2.27	7.85 ± 1.96	13.7 ± 2.1	7.25 ± 1.81
	Fe	206 ± 31	143.0 ± 21.5	149 ± 22	180 ± 27	206 ± 31	151 ± 23
	Zn	56.9 ± 8.5	49.3 ± 7.4	48.0 ± 7.2	59.9 ± 9.0	53.2 ± 8.0	46.5 ± 7.0
	Co	0.576 ± 0.144	0.491 ± 0.123	0.457 ± 0.114	0.568 ± 0.142	0.488 ± 0.122	0.434 ± 0.109
	Mn	82.0 ± 12.3	63.7 ± 9.6	66.4 ± 10.0	57.8 ± 8.7	94.8 ± 14.2	55.5 ± 8.3
	Mo	2.96 ± 0.74	0.760 ± 0.190	0.670 ± 0.168	0.742 ± 0.186	0.677 ± 0.169	0.534 ± 0.134
	Cr	0.681 ± 0.170	0.583 ± 0.146	0.480 ± 0.120	0.629 ± 0.157	0.589 ± 0.147	0.463 ± 0.116
	Se	2.31 ± 0.58	1.25 ± 0.312	0.977 ± 0.244	0.604 ± 0.151	0.562 ± 0.141	0.485 ± 0.121
	B	167 ± 25	95.1 ± 14.3	75.8 ± 11.4	75.6 ± 11.3	70.2 ± 10.5	43.0 ± 6.44
Alkali metal and alkaline earth	K	3550 ± 532	3289 ± 493	3459 ± 519	3720 ± 558	3722 ± 558	2968 ± 445
	Ca	3111 ± 467	2800 ± 420	2912 ± 437	2995 ± 449	2636 ± 395	2289 ± 343
	Mg	788 ± 118	697 ± 105	767 ± 115	805 ± 121	846 ± 127	711 ± 107
	Na	848 ± 127	823 ± 123	862 ± 129	918 ± 138	836 ± 125	747 ± 112
	Ba	4.8 ± 1.2	3.78 ± 0.94	4.69 ± 1.17	4.04 ± 1.01	4.36 ± 1.09	3.07 ± 0.77
Toxic elemnts	As	3.0 ± 0.6	1.25 ± 0.25	0.788 ± 0.158	0.621 ± 0.124	0.546 ± 0.109	0.318 ± 0.064
	Cd	LLD	0.030 ± 0.006	0.0137 ± 0.0027	0.0167 ± 0.0033	0.0172 ± 0.0034	0.00798 ± 0.00160
	Ni	0.758 ± 0.152	0.568 ± 0.114	0.607 ± 0.121	0.626 ± 0.125	0.747 ± 0.149	0.439 ± 0.088
	Pb	0.5 ± 0.109	0.713 ± 0.143	0.472 ± 0.094	0.381 ± 0.076	0.5228 ± 0.1046	0.354 ± 0.071
Other elements	Be	LLD	0.0122 ± 0.0030	0.0090 ± 0.0023	0.0110 ± 0.0027	0.0106 ± 0.0026	LLD
	Sr	8.8 ± 2.2	8.13 ± 2.03	8.52 ± 2.13	9.13 ± 2.28	8.35 ± 2.09	7.34 ± 1.84
	Ti	3.76 ± 0.94	3.20 ± 0.80	2.56 ± 0.64	3.65 ± 0.91	4.21 ± 1.05	1.84 ± 0.46
	Tl	0.137 ± 0.034	0.303 ± 0.076	0.475 ± 0.119	0.228 ± 0.057	0.09 ± 0.02	0.0828 ± 0.0207
	Al	118 ± 18	124 ± 19	106 ± 16	138 ± 21	164 ± 25	74.0 ± 11.1
	V	7.54 ± 1.88	6.88 ± 1.72	7.31 ± 1.83	7.73 ± 1.93	8.27 ± 2.07	6.49 ± 1.62

*LLD* lower limit of detection

#### Animals, housing

**Fig. 1:**
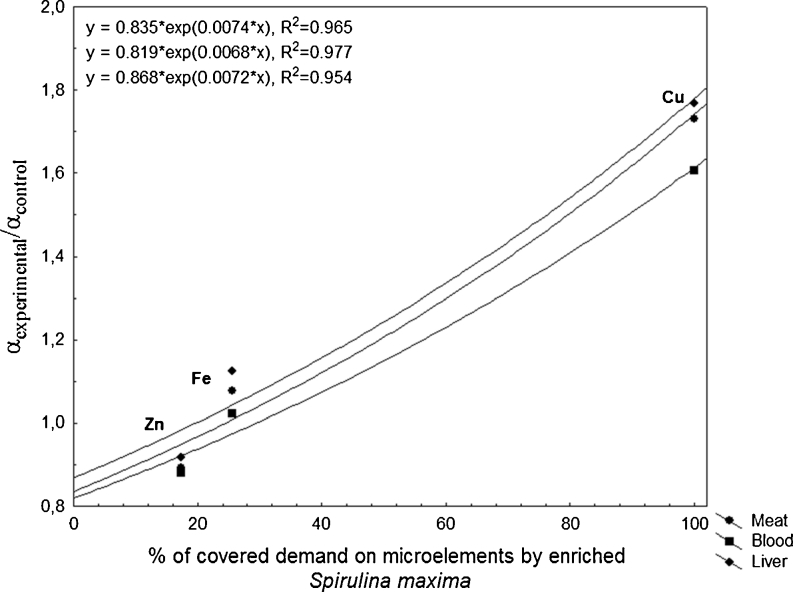
Correlation between ratio of *α*_experimental_ and *α*_control_ and % of covered demand on microelements by enriched *Spirulina maxima*

Dewormed (Dectomax® or Ivomec®) piglets (Big White Polish/Polish White Zwisloucha, dams × Hampshire/Pietrain) (24 pigs, 20.9 ± 2.2 kg) were randomly divided into two groups: 12 pigs in the control group and 12 in the experimental group. The three different feed compositions according to the different nutritional requirements for growth of the animals were used. Piglets in rearing phase (20–40 kg) were fed with the standard starter feed mixture, growers during the first period of fattening (40–65 kg) were fed with the standard grower feed mixture, and finishers in the second period of fattening (65–105 kg) were fed with the standard finisher feed mixture. Nutritional value of feed in specific periods of feeding is presented in Table 1–4. At the end of experiments, after swine reached their final weight, ten randomly chosen porkers were killed to obtain liver and meat (Fig. 1). Slaughter procedure was carried out in the slaughterhouse with the required permits and according to Minister of Agriculture and Rural Development dated 02/04/2004 by the persons entitled to professional slaughter and acceptable methods of slaughter and killing of animals (Polish Journal of Laws 2004.70.643). Approved procedure involves use of electronarcosis and exsanguination of pigs.

The study was performed in individual rearing pens with controlled microclimate (16–18 °C). Feed and water were available semi ad libitum. After the 21st day of feeding with grower feed, six porkers from each of group were separated for 7 days in individual cages and fed with the same feed. The first three 3 days were treated as the preliminary step. In the next 4 days, urine and feces were collected from each animal. Every morning, the amount of not consumed feed was recorded.

#### Sampling

The feeding experiment was conducted for 87 days and was divided into three series: starter (26 days), grower (31 days), and finisher (30 days), respectively (Fig. 1). After each series, each animal was weighed. On the 87th day, blood was collected. After separation, the concentration of microelements in serum was determined. Blood was sampled from the jugular vein. Before sampling blood, heparin was added to the samples in order to prevent blood coagulation. Muscle (longissimus dorsi muscle) and liver samples were homogenized. All samples with the exception of feed were kept in the freezer for multi-elemental analysis.

### Analytical methods

The appropriate mass of biological sample (feed—0.5 g, microalgae biomass—0.5 g, blood—4.0 g, meat (muscle)—0.5 g, urine—4.0 g, feces—0.5 g, liver—0.5 g) materials was digested with 5 mL concentrated—65 % (m m^−1^) HNO_3_ suprapur grade from Merck in Teflon vessels (microwave oven Milestone MLS-1200). After mineralization, all samples were diluted to 50 mL. Inductively coupled plasma–optical emission spectrometer with ultrasonic nebulizer (Varian VISTA-MPX ICP-OES, Australia) was used to determine the concentration of elements in algae and in all digested and diluted biological samples, in the Chemical Laboratory of Multielemental Analyses at Wroclaw University of Technology, which is accredited by ILAC-MRA and Polish Centre for Accreditation according to PN-EN ISO/IEC 17025.

### Calculations and statistical analyses

Shapiro–Wilk test was used to ensure that the data being used had a normal distribution. Levene's test and Brown–Forsythe test were used to assess the equality of variances in different samples. Significance of differences between the groups was examined with *U* Mann–Whitney (when the distribution was not normal), Welch (for data that have normal distribution and unequal variance), and *t* test (for data that have normal distribution and equal variance). Three levels of statistical significance were taken into account—at 0.1, 0.05, and 0.001. Statistical significance at *P* < 0.1 was regarded as a "trend", while 0.05 and 0.001 showed statistical significance of differences. The arithmetic mean values, standard deviations (SD), and *t* tests were carried out with the use of computer software *Statistica* ver. 9.0.

Feed conversion ratio (FCR) was calculated as the average of amount of feed ingested during the experiments divided by weight gain. A new coefficient *α* was defined to consider the real calculated (from ICP-OES analysis of samples) content of microelements in the feed which considers various doses of microelements in feed and enabled a comparison of bioavailability and was calculated according to the Eq. 1. This parameter is an indirect measure of availability of microelements.

$$ \alpha = \frac{{{\mathrm{BM}} \cdot {\mathrm{EC}} \left( {{\mathrm{blood}, \mathrm{meat} ~, \mathrm{or} ~ \mathrm{liver}}} \right)}}{{{\mathrm{APF}}{{\mathrm{I}}_{\mathrm{Starter}}} \cdot {\mathrm{E}}{{\mathrm{C}}_{\mathrm{Starter}}} + {\mathrm{APF}}{{\mathrm{I}}_{\mathrm{Grower}}} \cdot {\mathrm{E}}{{\mathrm{C}}_{\mathrm{Grower}}} + {\mathrm{APF}}{{\mathrm{I}}_{\mathrm{Finisher}}} \cdot {\mathrm{E}}{{\mathrm{C}}_{\mathrm{Finisher}}}}} $$

Apparent absorption defined as total intake minus feces excretion of the element, was calculated as the difference between intake and total feces excretion divided by intake (Ammerman 1995).

## Results

Pigs performance and animal health were very good in both groups throughout the experiment. No pigs were excluded from the experiment.

### Average feed intake, average weight gain and feed conversion ratio

The production performance over all periods is summarized in Table 5. No statistically significant differences were observed. Mineral supplementation in the form of enriched biomass of *Spirulina* did not affect the organoleptic properties of feed and its utilization (Table 5).

**Table 5:** Average production yields in different periods of feeding experiments: starter, grower, finisher

		Control group		Experimental group		Increase (↑)/decrease (↓)%	*P* values
		Mean	SD	Mean	SD		
Starter	BW (kg)						
	Start^a^	20.8	2.16	21.0	2.33	↑ 0.760	0.864
	End^a^	45.3	2.33	45.6	2.65	↑ 0.644	0.778
	APWG (kg)^a^	24.5	1.91	24.6	1.57	↑ 0.545	0.854
	ADWG (g)^a^	942	73.3	947	60.5	↑ 0.531	0.857
	APFI (kg)^c^	49.0	1.08	48.1	2.19	↓ 1.77	0.356
	ADFI (kg)^c^	1.88	0.040	1.84	0.087	↓ 2.21	0.225
	FCR^a^	2.01	0.162	1.96	0.153	↓ 2.45	0.452
Grower	BW (kg)						
	Start^a^	45.3	2.3	45.6	2.7	↑ 0.644	0.778
	End^a^	76.0	2.2	75.5	4.2	↓ 0.614	0.739
	APWG (kg)^a^	30.7	1.8	30.4	2.8	↓ 1.086	0.734
	ADWG (g)^a^	987	60	982	90	↓ 0.557	0.862
	APFI (kg)^c^	81.4	3.8	80.5	4.4	↓ 1.065	0.611
	ADFI (kg)^c^	2.62	0.12	2.59	0.14	↓ 1.340	0.615
	FCR^a^	2.65	0.07	2.66	0.26	↑ 0.567	0.848
Finisher	BW (kg)						
	Start^a^	76.0	2.2	75.5	4.2	↓ 0.614	0.740
	End^a^	107	3	105	5	↓ 1.84	0.256
	APWG (kg)^a^	31.1	2.0	29.6	3.5	↓ 4.85	0.210
	ADWG (g)^a^	1036	68	986	115	↓ 4.85	0.210
	APFI (kg)^c^	94.9	4.2	94.8	3.7	↓ 0.158	0.273
	ADFI (kg)^c^	3.17	0.16	3.16	0.12	↓ 0.315	0.273
	FCR^a^	3.07	0.21	3.25	0.46	↑ 6.03	0.227
Whole period	BW (kg)						
	Start^a^	20.8	2.2	21.0	2.3	↑ 0.760	0.864
	End^a^	107	3	105	5	↓ 1.84	0.256
	APWG (kg)^a^	86.3	3.9	84.1	5.4	↓ 2.47	0.279
	ADWG (g)^a^	991	45	967	62	↓ 2.47	0.279
	APFI (kg)^c^	225	8	223	6	↓ 0.902	0.273
	ADFI (kg)^c^	2.59	0.09	2.57	0.07	↓ 0.902	0.482
	FCR^a^	2.62	0.10	2.66	0.17	↑ 1.85	0.412

*ADFI* average daily feed intake, *APFI* average periodical feed intake, *ADWG* average daily weight gain, *APWG* average periodical weight gain, *BW* body weight

^a^ *t* test

^b^Cochran–Cox test

^c^Mann–Whitney test

### Concentration of microelements in collected samples

The average element concentrations in all collected samples were presented in Table 6. No statistically significant differences between the control and experimental group were observed in concentration of microelements in blood (Fig. 6). In meat, Cr concentration increased by 28 % (*P* < 0.05). Additionally, concentration of Cu, Mn, and Se increased by 18, 25, and 23 %, respectively (Table 6). In liver, a statistically significant decrease of Zn and Se was found, by 16 % (*P* < 0.1) and 95 % (*P* < 0.1), respectively (Table 6). At the same time, an increase of Cr by 54 % was observed. The content of Cu, Fe, Co, Mn, Cr, and Se in feces was lower than in the control group by 63 % (*P* < 0.001), 8 % (Ns), 9.5 % (*P* < 0.1), 5.3 % (Ns), 8.5 % (*P* < 0.05), and 19 % (Ns), respectively (Table 6). Only the content of Zn was higher than the control group by 19 % (*P* < 0.05). At the same time, the concentration of microelements excreted with urine was higher than the control group: 23 % (Ns) of Cu, 19 % (Ns) of Fe, 340 % (*P* < 0.001) of Zn, 72 % (*P* < 0.05) of Co, 190 % (*P* < 0.05) of Mn, 145 % (*P* = 0.1) of Cr and 336 % (*P* < 0.05) of Se (Table 6).

**Table 6:** The concentration (mg kg^−1^) (mean ± SD) of macro- and microelements in collected samples in control (C) and experimental (E) group

Group		Microelements							Macroelements					
		Cu	Fe	Zn	Co	Mn	Cr	Se	K	Ca	B	Mg	Na	Ba
Blood (*n* = 12)	C	0.841 ± 0.143	217 ± 31	2.21 ± 0.17	0.00134 ± 0.00156	0.0126 ± 0.0033	0.0507 ± 0.0071	0.049 ± 0.0505	971 ± 90	50.5 ± 6.8	359 ± 51	22.8 ± 2.8	656 ± 95	0.033 ± 0.0092
	E	0.892 ± 0.131	202 ± 13	2.14 ± 0.39	0.0011 ± 0.00147	0.0112 ± 0.0026	0.0477 ± 0.0023	0.0258 ± 0.0303	934 ± 58	45.9 ± 4.9	325 ± 7	22.8 ± 1.4	668 ± 65	0.0174 ± 0.0050
	Increase/decrease	6.06 % ↑	6.91 % ↓	3.16 % ↓	17.9 % ↓	11.1 % ↓	5.92 % ↓	47.4 % ↓	3.81 % ↓	9.11 % ↓	9.47 % ↓	=	1.82 % ↑	47.3 %↓
	*P* values	0.917	0.347	0.251	0.754	0.458	0.403	0.405	0.459	0.259	0.185	0.991	0.174	0.0105
Meat (*n* = 20)	C	0.76 ± 0.095	7.83 ± 1.72	25.6 ± 4.1	0.00342 ± 0.00814	0.136 ± 0.016	0.104 ± 0.023	0.0973 ± 0.1235	5990 ± 630	221 ± 35	1422 ± 94	366 ± 46	458 ± 79	0.13 ± 0.100
	E	0.823 ± 0.435	7.58 ± 2.75	23.4 ± 8.4	0.00342 ± 0.00814	0.17 ± 0.071	0.134 ± 0.039	0.12 ± 0.184	5572 ± 1972	185 ± 87	1409 ± 464	336 ± 118	442 ± 158	0,109 ± 0,088
	Increase/decrease	18.3 % ↑	3.19 % ↓	8.59 % ↓	=	25 % ↑	28 % ↑	23.3 % ↑	6.98 % ↓	16.3 % ↓	0.914 % ↓	8.19 % ↓	3.49 % ↓	16.2 % ↓
	*P* values	0.821	0.880	0.496	0.151	0.169	0.0343	0.757	0.820	0.0124	0.0588	0.820	0.545	0.545
Urine (*n* = 12)	C	0.0812 ± 0.0238	1.92 ± 0.72	1.32 ± 0.49	0.00935 ± 0.00467	0.124 ± 0.034	0.0191 ± 0.0098	0.0135 ± 0.0162	1081 ± 214	137 ± 33	213 ± 38	87.8 ± 24.4	210 ± 41	0.033 ± 0.0092
	E	0.0999 ± 0.0190	1.56 ± 0.83	5.79 ± 0.89	0.0161 ± 0.0025	0.36 ± 0.226	0.0467 ± 0.0497	0.0588 ± 0.0284	1645 ± 317	103 ± 45	353 ± 37	122 ± 16	321 ± 56	0.0174 ± 0.0050
	Increase/decrease	23.03 %↑	↓18.70 %	338 %↑	72.20 %↑	190↑	145↑	336↑	52↑	25↓	↑66	↑39	↑53	47↓
	*P* values	0.165	0.434	0.000001	0.0107	0.0298	0.100	0.00687	0.00473	0.173	0.000072	0.0170	0.00293	0.775
Feces (*n* = 12)	C	247 ± 49	4274 ± 934	1211 ± 107	7.18 ± 0.71	642 ± 51	8.94 ± 0.37	1.97 ± 3.11	8191 ± 1000	17994 ± 1211	LLD	6106 ± 560	4158 ± 768	80.5 ± 9.0
	E	90 ± 3	3930 ± 1881	1445 ± 157	6.5 ± 0.54	608 ± 26	8.22 ± 0.68	1.6 ± 2.50	8807 ± 1550	18365 ± 1251	LLD	5883 ± 267	3948 ± 1057	50.5 ± 2.2
	Increase/decrease	63.5↓	8.05↓	19.3↑	9.47↓	5.30↓	8.05↓	18.8↓	7.52↑	2.0↑6	–	3.65↓	5.05↓	37.27↓
	*P* values	0.000014	0.699 s	0.0131	0.0894	0.168	0.0437	0.873	0.522	0.796	=	0.787	0.702	0.000012
Liver (*n* = 12)	C	34 ± 13.3	733 ± 201	334 ± 57	1.13 ± 1.00	9.45 ± 1.43	0.305 ± 0.273	5.58 ± 8.71	8367 ± 222	208 ± 16	LLD	503 ± 14	2480 ± 263	0.24 ± 0.083
	E	33.7 ± 19.0	650 ± 144	280 ± 71	1.13 ± 1.28	10.2 ± 1.70	0.469 ± 0.294	0.27 ± 0.81	8558 ± 316	331 ± 101	LLD	502 ± 14	2351 ± 189	1.35 ± 1.06
	Increase/decrease	0.88↓	11.3↓	16.2↓	0.00	7.94↑	53.8↑	95.2↓	2.28↑	59.13↑	–	0.20↓	5.20↓	462.↑
	*P* values	0.627	0.329	0.0850	0.996	0.314	0.239	0.085	0.156	0.000349	=	0.855 s	0.247	0.000349

The addition of microelements bound with the biomass of *Spirulina* caused significant increase of macronutrients excretion in urine, 52 % (*P* < 0.05) of K, 66 % (*P* < 0.001) of B, 39 % (*P* < 0.05) of Mg, and 53 % (*P* < 0.05) of Na. Additionally, 25 and 47 % decrease of Ca and Ba excretion, respectively, was observed. In feces, the content of Ba decreased by 37 %.

The content of Ca and Ba significantly increased in liver by 59 % (*P* < 0.01) and 460 % (*P* < 0.01), respectively. In meat, a decrease was observed for macroelement concentrations, but those differences were not statistically significant. In blood, Ba concentration decreased by 47 % (*P* < 0.05).

Coefficient *α* (Table 7) presents the degree of absorption of microelements from the experimental and control diet according to the real concentration of those elements in feed. The *α* coefficient was higher for copper in the treatment than the control, 73 % (*P* < 0.05) in blood, 60 % (*P* < 0.1) in meat, and 76 % (*P* < 0.05) in liver. Coefficient *α* for iron in blood and meat was slightly higher, and in the case of liver 11 % (*P* < 0.1) higher in experimental group. Apparent absorption (Table 8) for Cu was 30 % (*P* < 0.05) higher in experimental group, while for Zn it was lower by 16 % (*P* < 0.1) in experimental group.

**Table 7:** The coefficient *α*, for blood, meat, and liver samples

		Control	Experimental	*P* value
		Mean ± SD	Mean ± SD	
Blood	Cu	0.0238 ± 0.00404	0.0412 ± 0.0040	0.00794
	Fe	0.396 ± 0.057	0.427 ± 0.057	0.310
	Zn	0.156 ± 0.030	0.139 ± 0.030	1.00
Meat	Cu	0.968 ± 0.377	1.55 ± 0.377	0.0400
	Fe	1.34 ± 0.367	1.37 ± 0.367	0.796
	Zn	0.164 ± 0.027	0.145 ± 0.027	0.436
Liver	Cu	0.021 ± 0.003	0.038 ± 0.003	0.00150
	Fe	0.0143 ± 0.0031	0.016 ± 0.0031	0.0630
	Zn	0.166 ± 0.026	0.152 ± 0.0265	0.971

**Table 8:** The apparent absorption of Cu, Fe, and Zn

	Control	Experimental	*P* value
	Mean ± SD	Mean ± SD	
Cu	64.3 ± 7.6	83.7 ± 1.8	0.000119
Fe	61.4 ± 10.7	71.5 ± 15.7	0.211
Zn	63.4 ± 4.9	53.2 ± 6.7	0.0131

## Discussion

The reasons why absorption of Zn was lower might be: the antagonism with copper or supplementation was not suitable form (zinc was supplied mostly in inorganic form), or both. The best results were obtained for copper, because 100 % was supplemented in the form bound with *S. maxima* enriched by biosorption process. The additional factor that could result in low availability of zinc was strong antagonism Cu–Zn and Fe–Zn. In Fig. 1, the correlation between ratio of *α*_experimental_/*α*_control_ and % of covered demand on microelements by enriched *S. maxima* was presented*.* The ratio *α*_experimental_/*α*_control_ expresses absorption of microelement in experimental group in comparison with the control group. To reach better absorption of microelement, it is advised to introduce the microelements in biological form, such as *S. maxima* enriched with microelement via biosorption process.

This study investigated the ways to minimize the effects of excreted Cu on the environment. Intensive animal breeding is considered to be a serious obstacle to sustainable development. Cu and Zn are often oversupplied in animal diets because they are used as growth promoters or because large safety margins are applied as a result of low bioavailability. Consequently, manure is highly concentrated in these elements, which may concentrate in top soil and cause toxicity to plants and microorganisms (Jondreville et al. 2003). Absorption rates may range from 0–99.5 % depending on the source as well as a host of other factors. It is very important to reduce metals load in manure by lowering the influx through animal feed. Application of highly bioavailable, bio-metallic feed additives based on microalgal biomass is one of the means to limit this environmental risk. In this paper, it was shown that by utilization of new biological feed supplements based on *Spirulina* enriched with microelements by biosorption, it was possible to reduce by 60 % excretion of copper in feces. At the same time, no effect on production results (BW, ADWG, ADFI, FCR) was observed.

The new mineral feed additives based on microalgae biomass can compete with other biological forms of microelements currently used. There are four types of organic supplements with microelements available: amino acid complex, amino acid chelates, polysaccharide complex, and proteinate (Grela and Rudnicki 2007; Murphy 2009). The complexes and chelates of microelements with amino acids would be four times more concentrated than the microelements bound with the biomass of algae. On the other hand, it is better to use less concentrated material for better distribution of mineral feed additives in the feed. Additionally, literature reports that the use of traditional inorganic sources of micronutrients causes oxidation of vitamins (Marchetti et al. 2000) and perforation of digestive tract (Chowdhury et al. 2004).

Bio-metallic feed additives from microalgae with designed composition are proposed to be a solution to problems with increasing accumulated quantities of trace elements in the environment. Application of this kind of new generation of bio-metallic feed additives would supply microelements in a form highly bioavailable to animals and would reduce amount of microelements of transit character used as supplements in animal feed.

According to the Directive of Polish Ministry of Agriculture and Rural Development concerning the maximum acceptable levels of undesirable substances in animal feed is 2 mg kg^−1^ for arsenic, 10 mg kg^−1^ cadmium, and 1 mg kg^−1^ lead. The levels of toxic elements in the diet of the experimental and in the control group were below the acceptable levels.

## Abbreviations

ADFI: Average daily feed intake
APFI: Average periodical feed intake
ADWG: Average daily weight gain
APWG: Average periodical weight gain
BW: Body weight
EC: Element content/concentration
FCR: Feed conversion ratio
